# The early function of cortisol in liver during *Aeromonas hydrophila* infection: Dynamics of the transcriptome and accessible chromatin landscapes

**DOI:** 10.3389/fimmu.2022.989075

**Published:** 2022-12-01

**Authors:** Hucheng Jiang, Mengling Sun, Yanhua Zhao, Guoxing Liu, Liqiang Zhong, Hui Xue, Xiaohui Chen, You Zheng, Minghua Wang

**Affiliations:** Freshwater Fisheries Research Institute of Jiangsu Province, Nanjing, China

**Keywords:** channel catfish (*Ictalurus punctatus*), cortisol, *Aeromonas hydrophila*, RNA-seq, ATAC-seq

## Abstract

In China, channel catfish (*Ictalurus punctatus*) is an important aquaculture species; however, haemorrhagic disease (*Aeromonas hydrophila* induced disease) in these fish has caused tremendous economic loss due to high morbidity and mass mortality in the breeding industry. The role of cortisol in bacterial diseases, particularly in the acute phase, remains unclear. In this study, liver transcriptome (RNA-seq) and chromatin accessibility (ATAC-seq) analyses were employed to investigate the early functional role of cortisol in *Aeromonas hydrophila*-stimulated responses. Our experiments confirmed that *A. hydrophila* infection can initially significantly increase serum cortisol levels at 1 h after infection. At this time point, the increased serum cortisol levels can significantly regulate *A. hydrophila*-regulated genes by affecting both transcriptome and chromatin accessibility. Cross-analysis of RNA-seq and ATAC-seq revealed that a certain gene group (92 target_DEGs) was regulated at an early time point by cortisol. KEGG enrichment analysis revealed that the top three pathways according to target_DEGs were cancer, glutathione metabolism, and the Notch signalling pathway. The protein-protein interaction analysis of target_DEGs revealed that they may be primarily involved in cell proliferation, CD8^+^ T cell function, glutathione synthesis, and activation of the NF-κB signalling pathway. This suggests that after the emergence of immune stress, the early regulation of cortisol is positive against the immune response. It is possible that in this situation, the animal is attempting to avoid dangerous situations and risks and then cope with the imbalance produced by the stressor to ultimately restore homeostasis. Our results will contribute to future research on fish and provide valuable insight regarding the mechanism of immune regulation by cortisol and the study of bacterial haemorrhagic disease in channel catfish.

## 1 Introduction

The potential importance of neuroendocrine-immune interactions in the physiological regulation of immunity and brain function is clearly established (1, 2). As the lymphatic tissue of vertebrates is innervated by parasympathetic and sympathetic nerve fibres, this process is related to the stimulation and suppression of immune function (3). In mammals, the adrenal gland is an important endocrine organ. The equivalent tissue of the adrenal gland is located in the head kidney of fish. Interrenal tissue (corticosteroid-producing cells), chromaffin cells, haematopoietic cells, lymphocytes, and macrophages are mixed within this tissue (4). The hypothalamic-pituitary-interrenal tissue (HPI) axis in fish is equivalent to the hypothalamic-pituitary-adrenal (HPA) axis in mammals (5). When mammals are under immune stress, the HPA axis is activated, and cortisol is subsequently released. In fish, renal interstitial cells within the head kidney secrete cortisol during the activation of the HPI axis (6).

In mammals, the regulation of immunity by cortisol under stress has been characterised (7). In fish, the role of cortisol in the immune system has also been demonstrated to regulate tissue inflammatory responses (8), lymphocyte/macrophage proliferation (9), cell phagocytosis (10, 11), decreases in antibody production (12), and increases in humoral immune protein levels (13). Cortisol inhibits neutrophil apoptosis (14), but cortisol is not just inhibitory which higher numbers of granulosa cells are found in higher levels of cortisol (15). In terms of bacterial stress, studies suggest that cortisol has multiple roles in immune regulation. Cortisol may inhibit the synthesis and proliferation of specific immune cells, but it can still increase the phagocytic ability of specific immune cells and effectively fight pathogens (7). Bacterial stressors can be thought of as causing inhibitory or adverse responses, but part of the response from cortisol regulation can also be thought of as active or potentiating responses. All of this seems to suggest that those energy-intensive and slow biological processes such as cell synthesis and cell proliferation may be inhibited by cortisol, and other faster fundamental responses (such as phagocytic capacity) are actively regulated against stressors. There is hypothesis believe that behind these complex events, the regulation strategy of cortisol depends on the time course of stress and the kinds of stressor (7). Additionally, the immune response caused by cortisol may be related to the energy stored within the animal and the mobilisation of energy resources (7). Indeed, under acute stress in the liver tissue of fish, energy metabolism pathways are activated to thus support this view (16). With the development of omics technology, an increasing number of details have been reported in recent years (17–19). However, the underlying molecular mechanisms remain poorly understood, particularly in regard to the initiation of the immune response and transcriptional regulation.

As the global aquaculture industry has suffered devastating losses due to disease outbreaks, research examining the immune response between pathogenic microorganisms and hosts has received increasing attention. *Aeromonas hydrophila* (AH) is a pathogenic microorganism that exerts a serious impact on freshwater aquaculture. It is a zoonotic pathogenic bacterium that exists widely in nature (20). AH is a gram-negative bacterium that can induce haemorrhagic sepsis in fish, and the mortality rate after infection is extremely high, thus seriously affecting the development of global freshwater aquaculture (21). Channel catfish are fast-growing fish possessing tender meat and high economic value. In recent years, the channel catfish farming industry has been plagued by a number of diseases (22). Diseases caused by bacteria (such as AH) can cause high mortality, greatly reduce the survival rate of channel catfish, cause huge economic losses, and severely affect the healthy development of channel catfish culture (23). Although there are reports of AH in channel catfish (24) and even liver tissues (including transcriptome studies), The importance and necessity of cortisol in AH infecting channel catfish need to be further described. Studying the underlying molecular mechanisms is not only important for aquaculture but also for understanding immune regulation.

In this study, cortisol levels in serum of channel catfish were modified, and AH was co-injected. Fish liver tissue and cortisol have multifunctional roles (metabolism and immunity). The cortisol under AH stress was assessed in detail by combining the use of RNA-seq and high-throughput sequencing accessible chromatin analysis (ATAC-seq) on liver tissue to help understand the role of cortisol in the fish immune response and its molecular mechanism, and aimed to provide novel insights for the study of cortisol regulation mechanisms under AH stress.

## 2 Method and materials

### 2.1 Experimental animal and samples

Animals were handled according to the guidelines for the care and use of animals for scientific purposes as set by the Institutional Animal Care and Use Committee of Freshwater Fisheries Research Institute of Jiangsu Province legislation (FFRI-DW-2019-020), Jiangsu Province, China. The test animals were non-endangered and propagated artificially. Channel catfish were collected from the Yangzhong Aquaculture Base of the Freshwater Fisheries Research Institute of Jiangsu Province, China. The average body weight of the channel catfish was 50 ± 3 g (n=250). The fish were adapted at 25 ± 1.5°C in a circulating aquaculture system for three week. Fish were then sorted into experimental groups one week before the start of the experiment, and acclimated for one week until experimental treatment. They were used in experiments for the detection of serum cortisol (CTS) levels under different treatment conditions and transcriptome assays. The pathogenic bacteria *Aeromonas hydrophila* (AH10) were used for bacterial challenge tests, and these bacteria were supplied by the Aquatic Pathogen Collection Centre of the Ministry of Agriculture, Shanghai, China. *A. hydrophila* was cultured at 28°C in LB medium (Sangon Biotech, China). In this study, bacteria were cultured and harvested during the exponential phase. Bacterial dilutions were plated onto LB agarose plates to counting living bacteria.

### 2.2 Experimental treatments and sampling

Two independent treatment experiments were conducted in this study. The first experiment was used to evaluate the response level of serum cortisol under different treatment conditions and to obtain the time point of early significantly response of cortisol then used for sampling operations in second experiment. According to the results of the first experiment, we obtained the sampling time point (1h), and we then running the second experiment, sampling for transcriptome analysis, and ATAC-seq analysis.

For the first experiment, the reagents were prepared by dissolving powdered metyrapone (Mtrp; Sigma 856525, Sigma Chemical Co., St. Louis, MO, USA) in absolute ethanol (200 mg/mL). The cortisol stock solution was prepared by dissolving powdered cortisol (cat. A610506, Shanghai Sangon Biotechnology Co., Ltd., Shanghai, China) in absolute ethanol (5 mg/mL). The reagents are injected intraperitoneally into the fish. The fish were divided into four treatment groups as follows: 1) the control group was the treatment group that receiving 0.1 mL of phosphate buffered saline (PBS) (0.01 mL absolute ethanol + 0.09 mL PBS); 2) the CTS group was the treatment group receiving 0.1 mL of cortisol solution (0.01 mL absolute ethanol containing 50μg cortisol + 0.09 mL PBS). Each experimental fish was injected in 1μg cortisol/g fish weight, the dosage was adjusted according to previous studies (4, 25, 26); 3) the cortisol inhibitor group was the treatment group that receiving 0.1 mL of cortisol inhibitor solution (0.01 mL of absolute ethanol containing 2 mg Mtrp + 0.09 mL PBS). Each experimental fish was injected in 40μg Mtrp/g fish weight, the dosage was adjusted according to previous studies (27, 28); 4) the *A. hydrophila* (AH) group was the treatment group that receiving a resuspension in PBS of 0.1 mL AH (0.01 mL absolute ethanol + 0.09 mL AH solution [2.6×10^6^ CFU/mL in PBS]). One tank (1.5m×1.5m×1.2m) per group contains 40 fish. The sampling times were set to 0h, 5 min, 1h, 2h, 4h, 8h, 12h, 24h, and 48h after intraperitoneal injection. After anaesthesia with tricaine mesylate (MS222, Sigma E10521, Sigma Chemical Co., St. Louis, MO, USA), whole blood was sampled with a needle and a syringe that have been previously prepared with heparin. Whole blood was centrifugated for separating the serum (1000×g, 10 min, 4°C), then serum was immediately frozen in liquid nitrogen until for cortisol level measurement. The radioimmunoassay (Automatic Radioimmunoassay System, XH6080, Xi’an Nuclear Instrument Factory) was used to measure cortisol content which customised by the Beijing Northern Institute of Biotechnology.

For the second experiment, the adapted fish were divided into four groups as follows: 1) the control group where each fish receiving 0.1 mL PBS (0.01 mL absolute ethanol + 0.09 mL PBS); 2) the Mtrp injected group where each fish receiving 0.1 mL of cortisol inhibitor solution (0.01 mL of absolute ethanol containing 2 mg of Mtrp + 0.09 mL PBS); 3) the CTS + AH injected group where each fish receiving 0.1 mL solution (0.01 mL of absolute ethanol which containing 50μg cortisol + 0.09 mL of AH solution which 2.6×10^6^ CFU/mL in PBS); 4) the Mtrp + AH injected group where each fish receiving 0.1 mL solution (0.01 mL of absolute ethanol which containing 2 mg Mtrp + 0.09 mL AH solution which 2.6×10^6^ CFU/mL in PBS). The reagents are injected intraperitoneally into the fish. One tank (1.0m×1.0m×0.8m) per group contains 8 fish. The sampling time was set to 1h after the fish were injected into their abdominal cavities. Liver tissue and whole blood was sampled after anaesthesia with tricaine mesylate (MS222, Sigma E10521, Sigma Chemical Co., St. Louis, MO, USA). Both serum separation and serum cortisol measurement protocol are the same as the first experiment. Liver tissue immediately frozen in liquid nitrogen for total RNA extraction that was followed by RNA-seq and ATAC-seq determination.

### 2.3 RNA-seq library preparation, sequencing, and analysis

RNAiso Plus (Takara, Dalian, China) was used to extract RNA according to the manufacturer’s protocol. A Nanodrop 2000C spectrophotometer (Nanodrop Technologies, USA), 1.2% agarose gel electrophoresis, and Agilent 2100 gel imaging system (Tianneng, Shanghai, China) were used to determine the quantity and quality of RNA in each sample. For high-purity RNA, the ratio of A260/A280 was considered to be between 1.8-2.1, and the brightness of 28S:18S RNA in the gel image is considered to be in close proximity to 2:1. We enriched mRNA with DNase to remove DNA residues and to remove microRNA with magnetic beads containing oligos (dT). RNA samples exhibiting an RNA integrity number (RIN) >7 were subjected to subsequent analysis. The library was constructed using the SMARTer^®^ PCR cDNA synthesis kit (Takara, Dalian, China) according to the manufacturer’s instructions. The library was sequenced on the Illumina HiSeq 2000 sequencing platform, and paired-end reads of 150 bp were generated.

The original sequencing data contained linker information, low-quality bases, and unmeasured bases (represented by N), all of which could cause significant interference in subsequent information analysis. Therefore, these potential interfering data were removed using fine-filtering methods (Trimmomatic V0.32). The resulting data were valid data that are also known as clean reads. The clean read sequence after each sample quality control was evaluated using FastQC V0.11.9. Additionally, the software HISAT2 v2.1.0 was used for the clean reads that were aligned to the reference genome (accession number: GCF_001660625.1 (29) to thereby complete the assembly work for the second-generation sequences.

To detect the differences between samples within a given group, we analysed the gene expression of the samples in the group to confirm that the gene expression of the samples in the group was within an acceptable range. The RSEM v1.3.0 software was used to compare the results of the bow tie to obtain the number of readings per sample in each transcript and to perform FPKM (number of fragments per kilobase per million) conversion. Additionally, the expression levels of genes and transcripts were determined. The R language package DEseq2 was used to perform difference analysis between the experimental group and the control group, and the screening threshold was FDR (false discovery rate) <0.05, log2FC (fold change [condition 2/condition 1] for a gene) >1, or log2FC < -1. Principal component analysis (PCA), hierarchical clustering, and volcano plots were created using the psych, factoextra, reshape2, and ggplot2 packages in Rstudio.

### 2.4 ATAC-seq library preparation, sequencing, and analysis

Each treatment group utilized two biological replicates that were prepared in the same manner for RNA-seq, and the ATAC-seq library was constructed using the method described in “section 2.2”. Briefly, separate cells from each combined sample were used to obtain a single-cell suspension. The cells were then suspended in nuclear isolation buffer and washed repeatedly with nuclear wash buffer in accordance with standard nuclear isolation protocols. We used 40,000 cell nuclei with Tn5 enzyme (Illumina) for the transposition reaction, and we recovered the transposed DNA fragments using a MinElute PCR Purification Kit (Qiagen). Then, the DNA fragments were amplified using high-fidelity PCR mix (NEB) with custom-barcoded primers for 5–10 cycles. The amplified product was recovered and purified using a MinElute PCR Purification Kit (Qiagen). Sequencing was performed on the Illumina Novaseq 6000 platform, and the sequencing strategy used was PE150.

Prior to read mapping, clean reads were obtained from the raw reads by removing the adaptor sequences (Trimmomatic V0.32). The clean reads were then aligned to reference genome sequences using the Burrows Wheeler Aligner (BWA) program. We calculated the fragment sizes for read pairs using a BAM file from paired-end sequencing. Several regions were sampled depending on the size of the genome and the number of processors to estimate the summary statistics of the fragment lengths. ChIP seq (MACS2 v2.1.2) model-based analysis was used to determine the ATAC-seq peak area of each sample. This method uses the bam file generated by the unique mapped reads as an input file and utilizes MACS2 software for callpeak with a cutoff qvalue < 0.05. Read distributions (from bigwig) across peaks are presented as an average plot (average of the read signals across all peaks). A deeptools tool was used for this analysis. The HOMER’s find Motifs Genome.pl tool was used for Motif analysis. For the visualisation of read count data, the bam files were first converted to bigwig files, and genome browser images were created using the Integrative Genomics Viewer (IGV) tools (30). The DNA sequence was extracted according to the peak file, and the sequence was compared with the Motif database to obtain the motif. To count the results of the annotations and to plot the distribution results, we used plotAnnoPie function in ChIPseeker. To perform a comprehensive analysis of ATAC seq and RNA-seq data, the gain or loss of ATAC-seq peaks within TSS ± 100 kb was analysed in all DEGs between pairwise comparisons. Peaks were annotated according to the annotate peak function in ChIPseeker. Additionally, DEGs containing different peaks in the three pairwise comparisons were identified, and functional enrichment analysis was subsequently performed.

### 2.5 GO and KEGG annotation and enrichments

Further KEGG and GO annotations were performed on the genes with NR annotation in the genome. The genome proteins were blasted against Swiss-Prot databases to obtain uniprot accession numbers using the Blastp algorithm with an E-value cutoff set at 1e^−5^. Meanwhile, the amino acid sequences of novel predicted genes were blasted against both the NR and Swiss-Prot databases to obtain refseq accession numbers and uniprot accession numbers using the Blastp algorithm with an E-value cutoff set at 1e^−5^. The UniProt accession number was used for annotation with Gene Ontology (GO). Additionally, the genome proteins were blasted against the Kyoto Encyclopaedia of Genes and Genomes (KEGG) databases (https://www.genome.jp/tools/kaas/). ClusterProfiler was applied to identify the significant GO categories, and the adjusted qvalue was used to correct the qvalue. We selected the significant terms (qvalue<0.05) according to enrichment analysis based on the up, down, and all differentially expressed genes to summarise the functions affected in the experiment. Pathway analysis was used to determine the significant pathways of the genes according to the KEGG database. We used ClusterProfiler to select the significant pathway, and the threshold of significance was defined according to qvalue and adjusted qvalue. We selected the significant pathway (qvalue<0.05) according to the enrichment analysis based on the up, down, and all differentially expressed genes to summarise the functions affected in the experiment.

### 2.6 Protein-protein interaction network construction

The functional interaction between proteins can provide context in regard to the molecular mechanism of the target_ DEG response. The PPI network of the DGEs was constructed using the Search Tool for the Retrieval of Interacting Genes (STRING v11.0, https://string-db.org/) database and subsequently visualised using Cytoscape v3.7.1 software. A confidence score >0.7 was set as the cut-off criterion.

### 2.7 Statistical analysis

Significant differences were analysed using Student’s *t*-test to determine significant differences between groups (*p < 0.05, ** *p* < 0.001). Data are presented as the mean ± SE.

## 3 Results

### 3.1 Serum cortisol level under treatments

**Figure 1:**
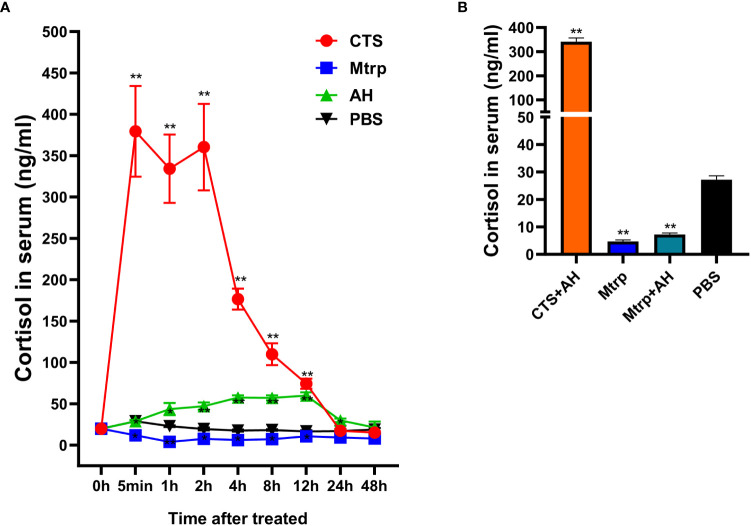
*Cortisol levels in the serum of channel catfish.* **(A)** First experiment, after reagents injection (Cortisol, CTS; Metyrapone, Mtrp; *A*. *hydrophila*, AH; and PBS) the cortisol level in serum (ng/ml) at 0h, 5 min, 1h, 2h, 4h, 8h, 12h, 24h, and 48h, respectively. **(B)** Second experiment, after reagents injection (Cortisol+ *A. hydrophila*, CTS+AH; Metyrapone, Mtrp; Metyrapone+*A. hydrophila*, Mtrp+AH; and PBS) the cortisol level in serum (ng/ml) at 1 hour. The value is the mean ± SD of five replicates. The t test was used to determine statistical significance. *P <0.05; ** P <0.01.

The results (**Figure 1A**) reveal that when cortisol is intraperitoneally injected, a significant increase in cortisol levels can be detected within a short time (< 5 min) in serum (*p <*0.001). The peak level of cortisol was maintained from 5 min to 2h, and the cortisol content then decreased until 48h to a level that was not significantly different compared to that of the control group. The cortisol inhibitor group also exhibited a significant decrease in cortisol levels at 5 min (*p <*0.05), and there was no significant difference compared to levels in the control group after 24 h. The cortisol level of the *A. hydrophila* injection group increased significantly after 1 h (*p <*0.05), and there was no significant difference compared to the levels in the control group after 48 h. the results from **Figure 1B** (second experiment) show that cortisol level in serum was significantly increasing (*p <*0.001) at cortisol + *A. hydrophila* co-injection group and significantly decreasing (*p <*0.001) at metyrapone injection and metyrapone + *A. hydrophila* co-injection group.

### 3.2 RNA-seq analysis

The RNA-seq raw data from the livers in all groups at 1h post-injection were greater than 6 billion bp clean reads pairs (**Supplementary Table 1A**). The clean reads Q20 values for all test samples were greater than 97%, and the Q30 values were greater than 93%. The RNA-seq raw data generated by the Illumina system were uploaded to the Gene Expression Omnibus (GEO database) NCBI (PRJNA793637). HISAT2 was used for mapping of clean reads to the reference genome, and the total mapped reads in each sample were greater than 91%, while the unique mapped reads were approximately 73% (**Supplementary Table 1A**). FPKM-based quantitative analysis was used to reveal the dynamic expression changes of all identified genes among the different injection groups (**Supplementary Figure 1A**).

**Figure 2:**
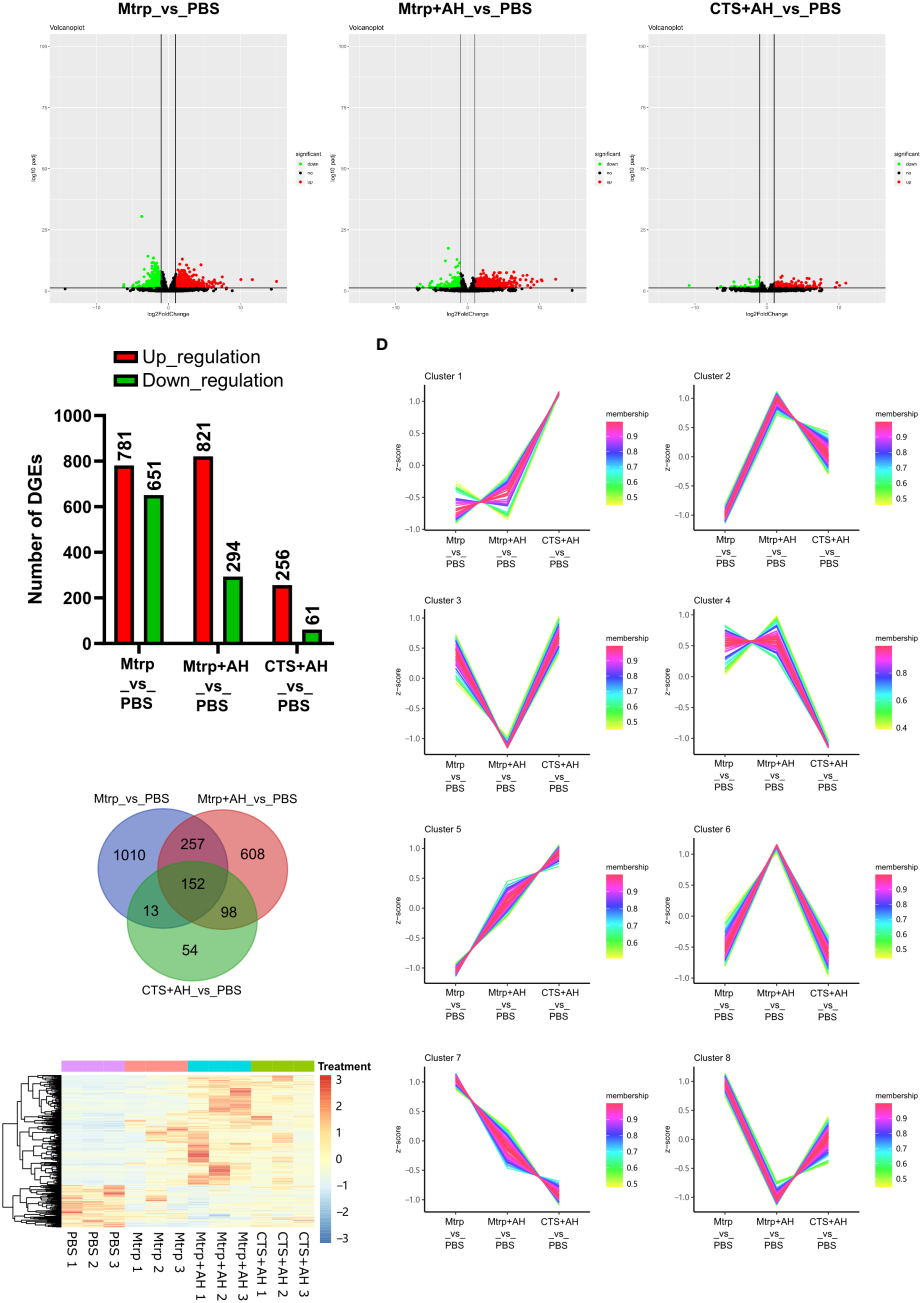
*RNA-seq reveals the transcriptome dynamics of the early immune response of cortisol after bacterial stimulation.* **(A)** Volcano plots presenting significantly up- and downregulated genes between Mtrp_*vs*_PBS, Mtrp+AH_*vs*_PBS, and CTS+AH_*vs*_PBS, respectively. **(B)** The number of differentially expressed genes (DEGs) for each pairwise comparison. **(C)** Venn diagram indicating the number of DEGs among three pairwise comparisons. **(D)** Heatmap of DEGs that are overlapped among three pairwise comparisons. **(E)** Selected candidate DEGs according to expression patterns of overlapped genes among three pairwise comparisons. Membership values indicate the degree to which a transcript belonged to this cluster.

After 1 h following Mtrp injection, a total of 1,432 genes were significantly different from the control group, including 781 genes that were upregulated and 651 genes that were downregulated (**Figures 2A, B** and **Supplementary Table 2**). After 1 h following Mtrp+AH injection, a total of 1,115 genes were significantly different from the control group, including 821 upregulated genes and 294 downregulated genes. After 1 h following CTS+AH injection, a total of 317 genes were significantly different from the control group, including 256 genes that were upregulated and 61 genes that were downregulated.

The venn diagram of the DEGs among the different treatments was constructed in **Figure 2C**, and the overlapping genes were clustered into eight groups based on the trend of the expression pattern (**Figure 2D**). A total of 615 candidate DEGs were identified in eight clusters (**Supplementary Table 3**, **Figure 2E**). GO enrichment analysis of candidate DEGs revealed that biological processes and molecular functions were more highly enriched. The number of genes that were enriched in the biological process was the largest, and the number of items that were enriched in the molecular function was the largest (**Supplementary Figure 2A**). KEGG enrichment analysis for candidate DEGs indicated that 14 pathways were significantly enriched (**Supplementary Figure 2B**). The top five pathways are proteasome, glutathione metabolism, antigen processing and presentation, apoptosis, and cell cycle.

### 3.3 ATAC-seq analysis

The ATAC-seq raw data from the livers in all groups at 1h post-injection were greater than 18 billion bp clean reads pairs were obtained (**Supplementary Table 1B**). The clean reads Q20 values of all test samples were greater than 97%, and the Q30 values were greater than 92%. The ATAC-seq raw data generated by the Illumina system were uploaded to the Gene Expression Omnibus (GEO database) NCBI (PRJNA793637). The total mapped reads in each sample were greater than 99%, and the unique mapped reads were approximately 89% (**Supplementary Table 1B**). Peak-based quantitative analysis was used in PCA analysis to reveal the accessible chromatin areas among the different injection groups (**Supplementary Figure 1B**).

**Figure 3:**
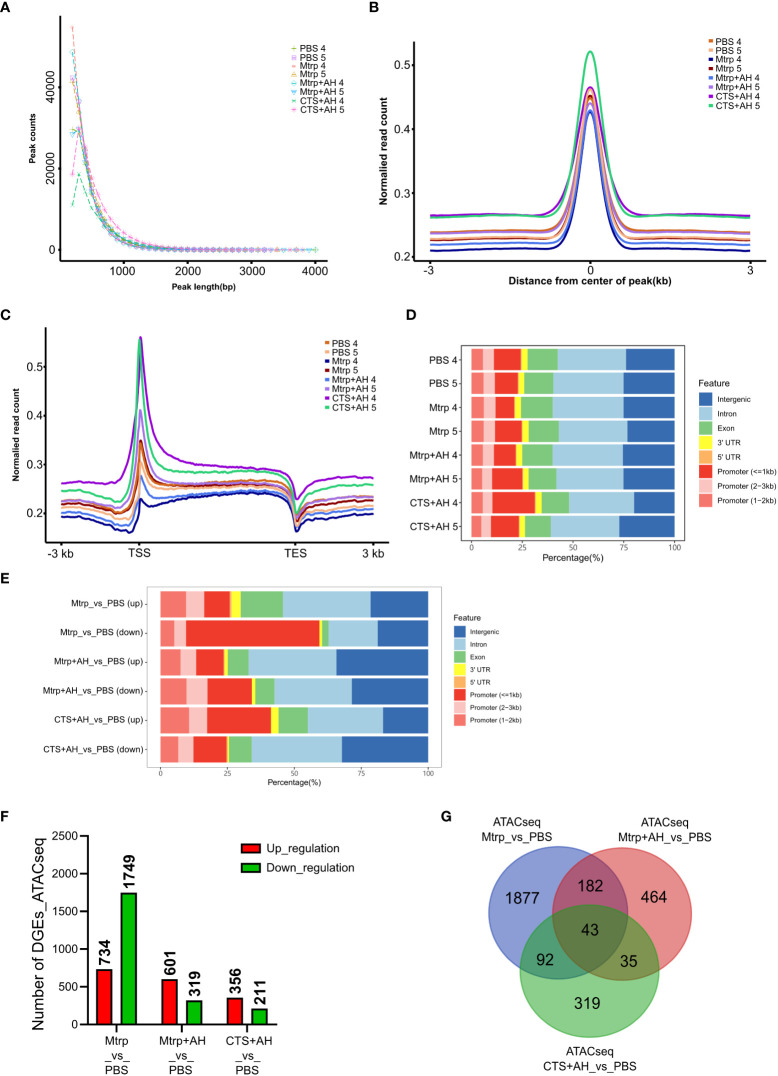
Quality estimation, peak call, and genome distribution of ATAC-seq reads during the early immune response process of cortisol after bacterial stimulation. **(A-C)** Distribution plot of sequencing reads from a representative ATAC-seq library across all genes. We normalized all genes according to their lengths and calculated the average ATAC-seq signals between TSS (–3 kb) and TES (+ 3 kb) for all genes after peak calling. **(D)** Number and genomic distribution of peaks identified by ATAC-seq in each sample. Genomic annotations included the promoter (<=1kb), promoter (2−3kb), promoter (1−2kb), exon, intron, 3’-UTR, 5’-UTR, and intergenic region, and the front region was regarded as the final annotation according to the above order if overlap occurred. **(E)** The number of differentially expressed genes (ATAC_DEGs) for each pairwise comparison (Mtrp_vs_control, Mtrp+AH_vs_control, and CTS+AH_vs_control). **(F)** Venn diagram indicating the number of ATAC_DEGs among three pairwise comparisons. **(G)** Number and genomic distribution of peaks identified by ATAC_DEGs in each sample.

In the context of different experimental treatments, the peak sizes exhibited certain distribution characteristics. The results revealed that most peak lengths were distributed below 1,000bp, and different treatment groups exhibited differences in peak length distributions and similarities between biological replicate samples (**Figure 3A**). The distribution of reads on the peak compared to those on the genome can be used to judge the quality of the experiment and the characteristics of the data. Deeptools software was used to calculate the average signal value of all peaks at each site in the normalised peak area and to draw these data into a graph. The results indicate that the reads for different samples are primarily enriched near the centre of the peak, thus implying that the signal in the peak area is more concentrated. Different treatment groups exhibited differences in the normalised read counts and similarities between biological replicate samples (**Figure 3B**). The average ATAC-seq signal distribution of all of the genes indicated that there was a strong signal in proximity to the TSS (**Figure 3C**), thus indicating that the majority of ATAC-seq reads were distributed around the TSS. Different treatment groups exhibited differences in the normalised read counts and similarities between biological replicate samples. As shown in **Figure 3D**, the quantitative analysis and peak annotation based on MACS2 demonstrate that the ratio of distribution of functional regions (intergenic region, intron, exon, 3’UTR, 5’UTR, and promoters) among the different treatment groups did not differ significantly.

We performed motif analysis on the difference peak (DP) to identify the binding sites of the corresponding transcription factors. Top10 motifs possessing P values of <0.05 are displayed in **Supplementary Table 4**. There were significant differences in the distribution of the functional regions (intergenic region, intron, exon, 3’UTR, 5’UTR, and promoters) of peaks in the treatment groups (**Figure 3E**). In the Mtrp injection group, DPs that were primarily distributed in promoters accounted for approximately 40%, and approximately 20% were distributed in the intergenic region. In the Mtrp+AH injection group, DPs that were primarily distributed in genes region accounted for approximately 40%, and the other ~30% were distributed in the intergenic region. In the CTS+AH injection group, the DP was primarily distributed in the gene region and accounted for approximately 45%, while the other ~25% was distributed in the intergenic region. The genes within 100kb downstream of the DP were annotated as target genes, and the results are presented in **Figure 3F** and **Supplementary Table 5**. After 1 h following Mtrp injection, a total of 2,483 genes were significantly different compared to the control group, including 734 genes that were upregulated and 1,749 genes that were downregulated. After 1 h following Mtrp+AH injection, 920 genes were significantly different compared to the control group, including 601 genes that were upregulated and 319 genes that were downregulated. After 1 h following CTS+AH injection, a total of 567 genes were significantly different from the control group, including 356 upregulated and 211 downregulated genes. The Venny plot reveals the overlap between different target genes in the different experimental treatment groups (**Figure 3G**).

### 3.4 Conjoint analysis of RNA-seq and ATAC-seq

**Figure 4:**
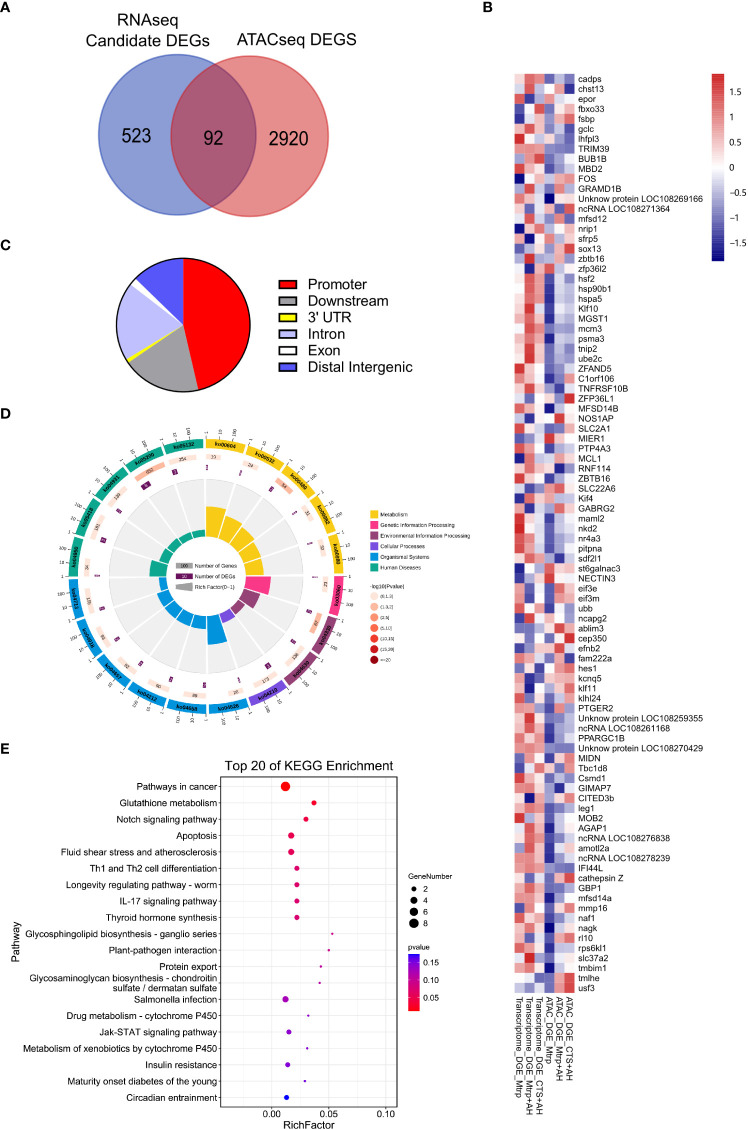
*The characteristics of target_DEGs in the overlap between ATAC_DEGs and candidate DEGs and the prediction of functions.* **(A)** Venn diagram indicates the number of target_DEGs between the ATAC_DEGs and candidate DEGs. **(B)** Heat map revealing target_DEGs in the RNA-seq and ATAC-seq samples. **(C)** The distribution of target_DEGs in the genome that associate the peak localization (ATAC-seq data). **(D)** KEGG pathways enriched by target_DEGs. The first circle: the classification of enrichment, where outside the circle is the coordinate ruler of the number of genes; the second circle: the number of background genes in the category and the Q value. More genes result in a longer bar, and smaller values are indicated by a redder colour; third circle: bar graph of total number of foreground genes; the fourth circle: the RichFactor value of each category (the number of foreground genes in the category divided by the number of background genes), where each cell of the background auxiliary line represents 0.1. **(E)** The top 20 KEGG pathways enriched by target_DEGs.

The venn diagram reveals the overlap genes (target_DEGs) which between the candidate DEGs from RNA-seq and the differential target genes from ATAC-seq (**Figure 4A**). The 92 target_DEGs were selected based on the overlap of RNA-seq candidate DEGs and ATAC-seq target genes of the various differential components (**Supplementary Figure 3**). **Figure 4B** presents the gene expression abundance and peak abundance of 92 target_DEGs in the RNA-seq and ATAC-seq samples. Additionally, the proportion of peak functional regions (intergenic region, intron, exon, 3’UTR, 5’UTR, and promoters) involved in target DEGs is presented in **Figure 4C**. Among these, this was primarily distributed in the promoter area (46%), and this was followed by the downstream (19%), intron (19%), and distal intergenic (13%) regions. KEGG was classified into six categories for display (**Figure 4D**). Pathways involved in human diseases were the most enriched (17 genes), and this was followed by organic systems (11 genes). The average RichFactor value for the metabolic pathway in each category was the highest. The analysis of the top 20 KEGG pathways enriched by target_DEGs revealed that the top three pathways with the most DEG enrichment were pathways in cancer, glutathione metabolism, and notch signalling (**Figure 4E**).

### 3.5 PPI analysis between target_DEGs

**Figure 5:**
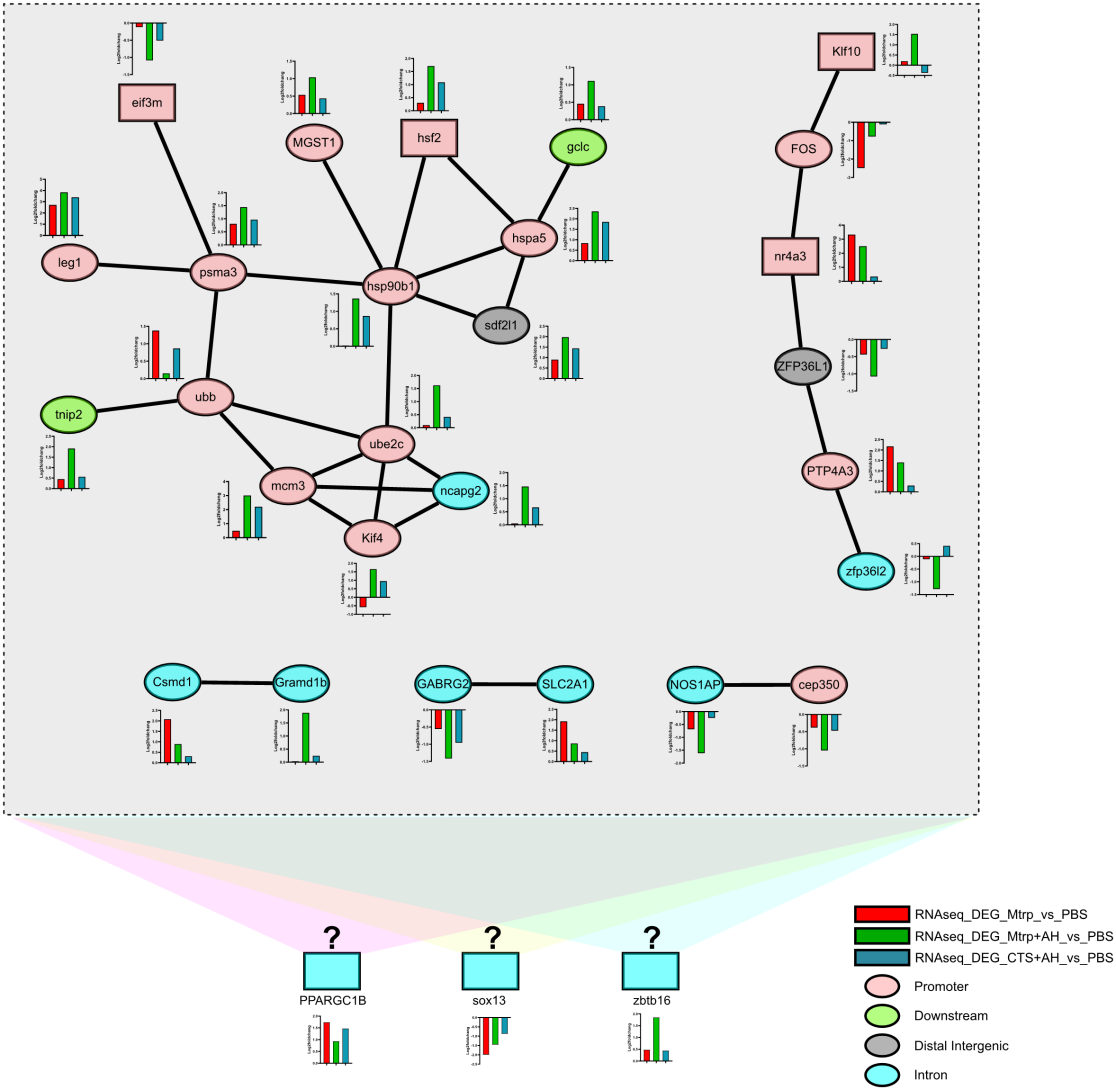
*The network diagram of the interaction between the target_DEGs based on the zebrafish protein-protein interaction database.* Rectangular boxes and ellipses represent genes for transcription factors and proteins, respectively. Colours in rectangles and ellipses represent the distribution of genes within the genome that associate the peak localization (ATAC-seq data). The different colours in the bar graph indicate the expression of genes in the transcriptome. Different coloured backgrounds indicate unproven potential TF-protein interactions.

Five protein-protein interaction networks in 92 target_DEGs was obtained (**Figure 5**, **Supplementary Table 6**). Among them, the interaction of 15 target_DEGs forms the largest interaction network that contains two transcription factors (eif3m and hsf2); however, the motif of these two transcription factors is not enriched in the ATAC-seq data. Almost all target_DEGs genes in this network were significantly upregulated in response to AH (Mtrp+AH_*vs*_Mtrp) and then significantly downregulated by CTS (CTS+AH_*vs*_Mtrp+AH) with the exception of the eif3m and ubb genes (their expression trends were significantly downregulated in response to AH and then significantly upregulated by CTS). In this network, all genes with the exception of gclc, trip2, and sdf2l1 were distributed in the promoter region (ATAC-seq data). Additionally, the interaction of six target_DEGs formed an interaction network that contained two transcription factors (Klf10 and nr4a3). Similarly, motifs combining these two transcription factors were not enriched in the ATAC-seq data. The remaining three networks were composed of two target_DEGs. With the exception of these 27 target_DEGs, 65 target_DEGs exhibited no interaction. As studies examining transcription factor binding to motifs in fish are rare, three transcription factors (PPARGC1B, sox13, and zbtb16) from target_DEGs are distributed in introns (ATAC-seq data) in addition to the transcription factors involved in the aforementioned networks that are considered to be potentially involved in CTS and the immune regulation process. The ATAC-seq profiles of these three genes exhibited variations among the different treatment groups (**Supplementary Figure 4**). These results suggest that altered gene expression levels may be correlated with dynamic changes in chromatin accessibility during early ovarian development.

## 4 Discussion

The mechanisms responsible for cortisol-regulated immunity remain poorly understood. In this study, transcriptomic and chromatin accessibility dynamics were assessed in channel catfish liver at 1h using cortisol inhibition and enhancement experiments. A cortisol response occurs during bacterial infection, thus implying that cortisol is involved in immune regulation. A number of details regarding the involvement of cortisol in regulating the immune response have also been reported (31–34). However, the acute immunoregulatory role of cortisol in the initial up-regulated short time period under bacterial stress remains to be further investigated. The experimental results in this study demonstrate (**Figure 1**) that serum cortisol becomes significantly up-regulated at 1h after AH infects the channel catfish, implying that the HPI axis modulates cortisol increases at 1 hour in response to bacterial stress. Mtrp treatment significantly reduced serum cortisol at 1h after treatment. Similarly, the serum cortisol level at 1h after CTS treatment was significantly increased, thus implying that the comparison between the treatments in this study (transcriptomic and chromatin accessibility dynamics) allowed us to explore to a large extent of the early behaviour of cortisol immunomodulation. Cortisol has been reported to play a regulatory role in physiological metabolism (35). Based on this background, liver tissue was selected (both immune tissue and an important organ of metabolism) for use in this study.

Recent advances in the ability of researchers to determine dynamic gene expression and chromatin accessibility changes in human and mouse liver tissues and cells using next-generation sequencing-based epigenomic techniques (e.g. RNA-seq, CHIP-seq, DNase-seq, and ATAC-seq) have contributed to the identification of key genes, cis-regulatory elements, and organism-specific regulatory systems (36–38). Understanding how gene regulatory networks are controlled by CTS in the immune system remains a long-standing challenge. Therefore, we integrated RNA-seq and ATAC-seq technologies to unravel the transcriptional networks regulating early immune regulation by CTS. By examining the number of differential gene responses (**Figure 2**), we observed that the number of genes responding to high levels of serum cortisol was far less than that in the group in which serum cortisol was inhibited. Additionally, in the case of low levels of serum cortisol, AH infection causes a greater amount of differential up-regulation of genes and fewer differentially downregulated genes. These results suggest that rising cortisol levels can significantly regulate certain AH-regulated genes. This also fits the role of cortisol in the rapid regulation of homeostasis (39). After performing cluster analysis on the expression trend of the DEGs (between the treatment group and the control group), we obtained a total of eight clusters that included 615 candidate DEGs. Their expression trends changed significantly in the presence of cortisol, thus suggesting that they may be the early regulatory gene of cortisol in the liver tissue. The functional characteristics (GO terms and KEGG pathway enrichment) of these 615 candidate genes revealed that the significantly enriched GO terms and KEGG pathway members were predominantly involved redox-related glutathione metabolism, the ubiquitin-proteasome pathway, antigen processing and presentation, apoptosis, and threonine endopeptidase activity. This also verified that cortisol is a key hormone in the fish stress response that can regulate a variety of physiological functions, including energy metabolism and the immune system (40).

The chromatin accessibility results between samples of each treatment group cryptically reveal differences in the distribution characteristics of peak counts and peaks on the chromosome (**Figures 3A-D**). The target gene analysis of differential peaks from the comparison between the treatment group and the control group highlighted the functional distribution characteristics of chromatin accessibility that is regulated by AH and CTS (**Figures 3E-G**). The number of genes in response to high levels of serum cortisol was less than that in the group in which serum cortisol was inhibited. This result was consistent with the RNA-seq results. Additionally, in the case of low levels of serum cortisol, AH infection results in less upregulation and downregulation of target genes. This result is slightly different compared to the RNA-seq results. The same ATAC-seq results also imply that rising cortisol levels can significantly regulate certain AH-regulated genes. The most highly differentiated peaks were distributed within the promoter region after Mtrp treatment; however, they decreased significantly after Mtrp+AH and CTS+AH treatments, thus suggesting that the increase in cortisol levels significantly regulates gene transcription expression.

Our results revealed that the smallest changes in both the liver transcriptome and chromatin accessibility occurred during the transition from CTS+AH_*vs*_PBS, thus indicating that the increase in CTS levels can restore some AH-regulated genes to the control level, while other genes are regulated to participate in the response that can be mapped to chromatin accessibility (**Figures 4A, B**). These results indicate that transcriptional activity of target_DEGs (the overlap between the RNA-seq candidate DEGs and ATAC-seq difference peak target genes) is strongly associated with the accessibility of functional genomic regions that are finely tuned at the chromatin level, and this is consistent with previous findings in a range of vertebrate species (41–44). As revealed by ATAC-seq analysis (**Figure 4C**), the majority (~80%) of CTS-selective peaks were present in the promoter (~45%), intronic, downstream, exonic, and 3’UTR regions. Our data enhance the notion that transcriptional changes during CTS increases that occur under AH infection are primarily regulated by functional gene regions, particularly the promoter elements from the gene locus itself. KEGG pathway enrichment analysis revealed that these target_DEGs were predominantly enriched in pathways related to metabolism, organismal systems, and human diseases (**Figure 4D**). Specifically, pathways in cancer, glutathione metabolism, apoptosis, and Th1 and Th2 cell differentiation were the most significant (**Figure 4E**), thus indicating that the functional roles of these target_DEGs are mainly metabolism and immunity. The increase in CTS levels after AH infection is likely to function by regulating the transcription and expression of metabolism and immune-related genes (24, 35).

Among 92 target_DEGs, five protein-protein interaction (PPI) networks were obtained based on the zebrafish database (**Figure 5**). It should be noted that although two of the networks contain four transcription factors, the peaks they involve are distributed within the promoter region of the gene locus itself. Transcription factors typically bind near the transcription start site of genes to regulate the transcription of upstream and downstream genes (45, 46). Based on this, we speculate that the four transcription factors involved in these networks may not be the initiation transcription factors regulated by CTS. The motifs contained in the different peaks of the four transcription factor promoter regions may be connected to the initiating regulatory factors; however, the results of the motif analysis based on ATAC-seq indicate that there are no transcription factors that target and bind the motifs of the promoter regions of the above four transcription factors in the target_DEGs list. This implies that these networks may not be complete. Additionally, the regulatory factors combined in the exon and intron regions of the gene may affect the variable splicing behaviour of the gene (47, 48). Here, we considered several possible reasons for this observation: 1) genes change their expression possibly through the binding of different transcription factor combinations to equally accessible sites (49–51); 2) other undefined protein-protein interaction mechanisms may exist in zebrafish and fish; 3) other undefined transcription factors may exist in fish. Based on the above considerations, we identified three transcription factors in the target_DEGs list (the peaks involved were distributed in the intronic region). We hypothesised that they may participate in the regulation of networks. Further studies are required in the future to test this hypothesis.

Fifteen target_DEGs formed the largest network, and their gene expression trends were nearly identical (with the exception of eif3m and ubb). Specifically, AH significantly upregulated these genes in response to low levels of cortisol, and high levels of cortisol downregulated these genes (compared to the former). This implies that they may be the early gene group regulated by cortisol in liver tissue. The protein interaction group composed of ube2c, ncapg2, kif4, and mcm3 is related to cell proliferation. For ube2c that functions to disrupt the process of cell proliferation (52, 53), CTS can significantly downregulate its upregulated expression by AH. This result suggests that CTS may cause the proliferation of certain cells in the liver tissue at the initial stage of AH infection. This result was also reported by Ciliberti et al. Cortisol can cause sheep peripheral blood mononuclear cells to proliferate under an acute 24-hour emergency (54). Pagniello et al. reported that the proliferation of rainbow trout macrophages is regulated by cortisol (55). Associating the interaction between nr4a3 transcription factor and FOS gene based on the observation that nr4a3-deficient murine CD8+ T cells differentiate preferentially into memory precursor and central memory cells and also produce more cytokines (56), we speculate that these proliferated cells may contain liver memory CD8+ T cells. Additionally, MGST1 and gclc genes act to reduce glutathione (57) and glutathione feedback regulation (58), respectively, and appear near the interaction network connected by hsf2, hsp90b1, and hspa5. This suggests that CTS produces more oxidised glutathione in the liver tissue in the early stage of AH infection (59, 60). The increase in oxidised glutathione in the liver is undoubtedly a manifestation of the body attempting to actively resist AH infection (61). NF-κB signalling pathway is one of the important response pathways of channel catfish bacterial infection (62, 63). It should be noted that eif3m, as a transcriptional activator/repressor (64), played a role as a transcriptional inhibitor in our study and regulated the psma3 gene. Previous studies have observed that the psma3 gene is an important component of the 26S proteasome that is involved in the activation of the NF-κB signalling pathway (65), and it is also an important component of the 20S proteasome that is involved in the processing of class I MHC peptides (66). The psma3 gene negatively regulates the ubb gene that is involved in the activation of the NF-κB signalling pathway as one of the ubiquitination genes (65). Additionally, ubb negatively regulates the tnip2 gene that has been reported to inhibit the activation of the NF-κB signalling pathway (67). Based on the above results, we speculate that CTS accelerated the activation of the NF-κB signalling pathway in certain cells of the liver tissue in the early stage of AH infection. Interestingly, NF-κB signalling pathway was still the main response pathway in the genome-wide association study (GWAS) of Aeromonas septicemia disease even though there were differences in experimental treatments compare to this study (24). It is worth noting that our results appear to counter the discussion regarding the inhibitory regulation of cytokines from cortisol (68–70). Here, we considered a possible reason for this observation, where the duration regulation of cortisol on the time axis does not appear to be unidirectional (4, 54). Cortisol produced in response to stress has been reported in mammals to inhibit or enhance certain pathways of the immune response (71, 72), and that these responses are modulated (activated or inhibited) differently depending on the stressor (72). There is a hypothesis that differences in response strategies of cortisol in response to stressors may be related to the mobilization of stored energy and energy resources in animals (7). It is speculated that cortisol differences drive the immune system to resist and destroy invading pathogens, organize resources to avoid harmful challenges, and divert nutrients to support these activities. Of course, such an explanation requires further study to elucidate its mechanism in fish.

## Data availability statement

The datasets presented in this study can be found in online repositories. The names of the repository/repositories and accession number(s) can be found in the article/**Supplementary Material**.

## Ethics statement

The animal study was reviewed and approved by The Freshwater Fisheries Research Institute of Jiangsu Province Ethical Committee.

## Author contributions

HJ, MW, GL and HX planned and supervised the study. HJ performed the bioinformatic and comparative analysis. HJ, MS and YZhao performed the experimental work. HJ, MW, GL and HX analyzed the results and wrote the manuscript. HJ, LZ, XC and YZheng revised the manuscript according to the reviewers' comments. All authors contributed to the article and approved the submitted version.

## Funding

This study was supported by the Earmarked fund for Jiangsu Agricultural Industry Technology System [JATS (2022)411] and the China Agriculture Research System of MOF and MARA (CARS-46).

## Conflict of interest

The authors declare that the research was conducted in the absence of any commercial or financial relationships that could be construed as a potential conflict of interest.

## Publisher’s note

All claims expressed in this article are solely those of the authors and do not necessarily represent those of their affiliated organizations, or those of the publisher, the editors and the reviewers. Any product that may be evaluated in this article, or claim that may be made by its manufacturer, is not guaranteed or endorsed by the publisher.

## Supplementary material

Supplementary Figure 1
*Principal component analysis (PCA) of the mRNA transcriptome in the four libraries.* **(A)** ATAC-seq peaks from four libraries **(B)**. Three and two biological replicates from each of the three different treatment groups and the PBS control group were subjected to RNA-seq and ATAC-seq, respectively, and their respective PCA charts ranked the main components according to the amount of data variation.

Supplementary Figure 2
*GO and KEGG analysis of DEGs among all comparisons (Mtrp_vs_control, Mtrp+AH_vs_control, CTS+AH_vs_control).* The bubble plot and graph represent the top 20 GO categories of DEG-enriched biological processes among all comparisons (Mtrp_*vs*_control, Mtrp+AH_*vs*_control, CTS+AH_*vs*_control). The horizontal bar graph represents the top 20 KEGG pathways enriched by DEGs between all comparisons (Mtrp_*vs*_control, Mtrp+AH_*vs*_control, CTS+AH_*vs*_control).

Supplementary Figure 3 The number of DEGs according to the comparison of the ATAC-seq three-pair processing (ATAC_Mtrp_vs_control, ATAC_Mtrp+AH_vs_control, ATAC_CTS+AH_vs_control) dataset. The small Venn diagram indicates the number of target_DEGs between the ATAC_DEGs and candidate DEGs.

Supplementary Figure 4 Genomic view of ATAC-seq peaks near the DEG of three transcription factors among four different treatment groups (PBS, Mtrp, Mtrp+AH, and CTS+AH), including PPARGC1B, sox13, and zbtb16. Differences among the different treatment groups. ATAC-seq peaks are marked with black boxes. The yellow bar indicates the chromosome, and the red box indicates the position of the displayed peak on the chromosome.
