# Supplementary figures and images for: The early function of cortisol in liver during Aeromonas hydrophila infection: Dynamics of the transcriptome and accessible chromatin landscapes
Source: Front Immunol. 2022 Dec 1;13:989075. doi: 10.3389/fimmu.2022.989075 (PMC9751032; doi:10.3389/fimmu.2022.989075)

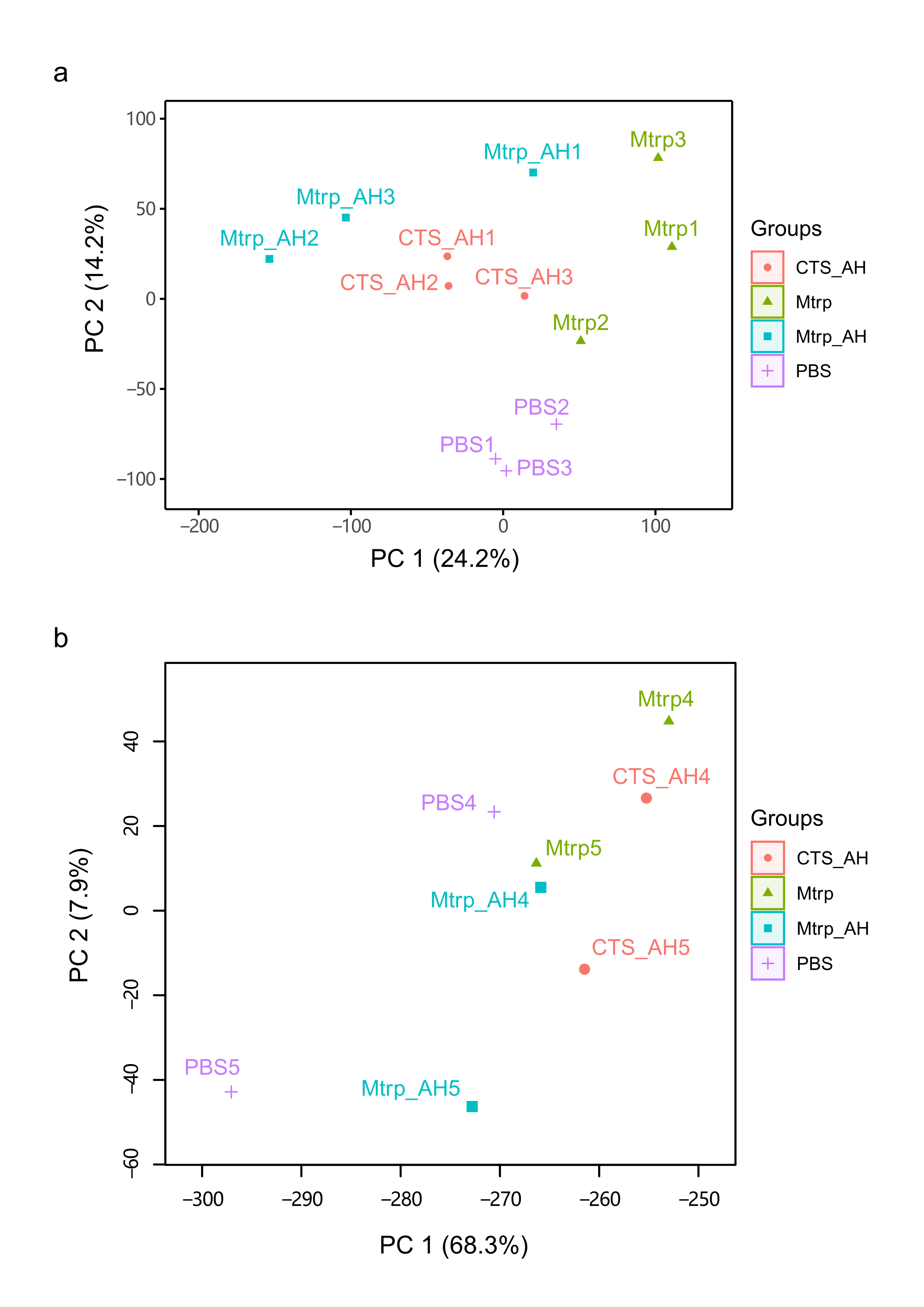

Supplement: Supplementary Figure 1 — Principal component analysis (PCA) of the mRNA transcriptome in the four libraries. (A) ATAC-seq peaks from four libraries (B). Three and two biological replicates from each of the three different treatment groups and the PBS control group were subjected to RNA-seq and ATAC-seq, respectively, and their respective PCA charts ranked the main components according to the amount of data variation. [file Image_1.tif]

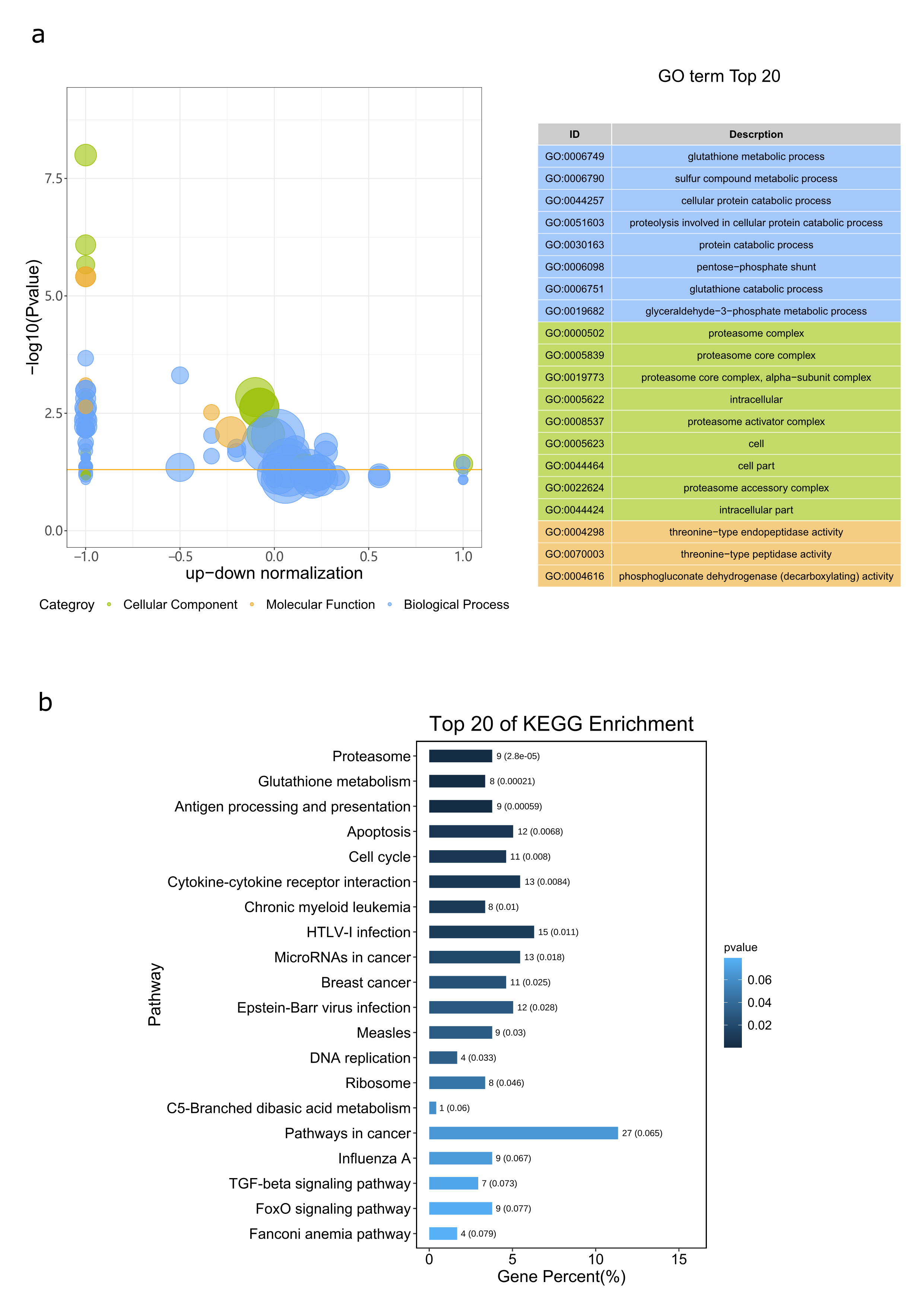

Supplement: Supplementary Figure 2 — GO and KEGG analysis of DEGs among all comparisons (Mtrp_vs_control, Mtrp+AH_vs_control, CTS+AH_vs_control). The bubble plot and graph represent the top 20 GO categories of DEG-enriched biological processes among all comparisons (Mtrp_vs_control, Mtrp+AH_vs_control, CTS+AH_vs_control). The horizontal bar graph represents the top 20 KEGG pathways enriched by DEGs between all comparisons (Mtrp_vs_control, Mtrp+AH_vs_control, CTS+AH_vs_control). [file Image_2.tif]

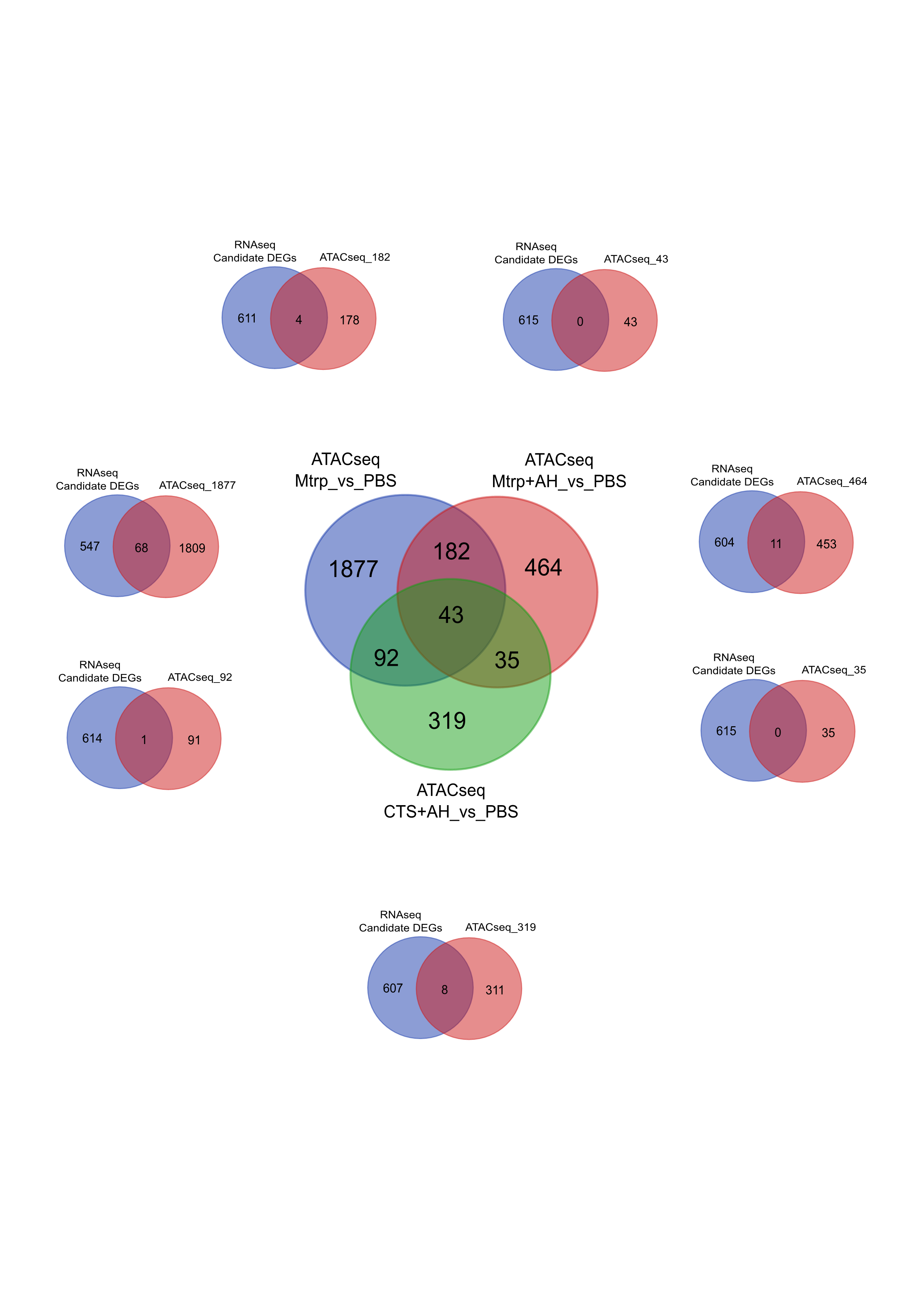

Supplement: Supplementary Figure 3 — The number of DEGs according to the comparison of the ATAC-seq three-pair processing (ATAC_Mtrp_vs_control, ATAC_Mtrp+AH_vs_control, ATAC_CTS+AH_vs_control) dataset. The small Venn diagram indicates the number of target_DEGs between the ATAC_DEGs and candidate DEGs. [file Image_3.tif]
