# Supplementary material for: The early function of cortisol in liver during Aeromonas hydrophila infection: Dynamics of the transcriptome and accessible chromatin landscapes
Source: Front Immunol. 2022 Dec 1;13:989075. doi: 10.3389/fimmu.2022.989075 (PMC9751032; doi:10.3389/fimmu.2022.989075)
Supplement: Supplementary file 4 [file Table_1.docx]

**Supplementary table 1a** Statistics of the Illumina sequencing data (RNAseq) and alignment the clean reads with reference genome

| **Sample** | **Number of raw bases (billion)** | **Number of raw reads** | **Q20 ratio (%)** | **Q30 ratio (%)** | **GC content (%)** | **Total mapped genome (%)** | **Unique mapped genome (%)** |  |
| --- | --- | --- | --- | --- | --- | --- | --- | --- |
|  |  |  |  |  |  |  |  |  |
| PBS-1 | 6.88 | 45896998 | 97 | 93 | 48 | 92.75 | 76.16 |  |
| PBS_2 | 6.54 | 43615216 | 97 | 93 | 48 | 93.49 | 76.76 |  |
| PBS_3 | 7.27 | 48465910 | 97 | 93 | 47 | 92.61 | 75.91 |  |
| Mtrp_1 | 9.79 | 65279220 | 97 | 94 | 49 | 93.21 | 75.68 |  |
| Mtrp_2 | 7.77 | 51807336 | 97 | 93 | 47 | 92.53 | 76.42 |  |
| Mtrp_3 | 6.35 | 42325872 | 97 | 93 | 47 | 92.22 | 74.39 |  |
| Mtrp+AH_1 | 7.11 | 47424646 | 97 | 93 | 47 | 92.49 | 75.14 |  |
| Mtrp+AH_2 | 8.64 | 57571504 | 97 | 93 | 47 | 91.63 | 74.39 |  |
| Mtrp+AH_3 | 7.01 | 46676766 | 97 | 93 | 47 | 92.33 | 75.28 |  |
| CTS+AH_1 | 8.08 | 53862366 | 97 | 93 | 47 | 92.81 | 75.73 |  |
| CTS+AH_2 | 7.83 | 52194582 | 97 | 93 | 47 | 91.89 | 74.23 |  |
| CTS+AH_3 | 7.06 | 47095068 | 97 | 93 | 47 | 92.11 | 73.11 |  |

**Supplementary table 1b** Statistics of the Illumina sequencing data (ATACseq) and alignment the clean reads with reference genome

| **Sample** | **Number of raw bases (billion)** | **Number of raw reads** | **Q20 ratio (%)** | **Q30 ratio (%)** | **GC content (%)** | **Total mapped genome (%)** | **Unique mapped genome (%)** |  |
| --- | --- | --- | --- | --- | --- | --- | --- | --- |
|  |  |  |  |  |  |  |  |  |
| PBS_4 | 21.98 | 146511020 | 97.93 | 93.5 | 43.5 | 99.2 | 90.9 |  |
| PBS_5 | 23.36 | 155740614 | 97.56 | 92.42 | 43.55 | 99.4 | 91.5 |  |
| Mtrp_4 | 23.8 | 158688272 | 98.09 | 94.03 | 44.12 | 99.4 | 89.4 |  |
| Mtrp_5 | 23.8 | 158634774 | 97.56 | 92.48 | 43.85 | 99.6 | 90.4 |  |
| Mtrp+AH_4 | 24.5 | 163318368 | 97.89 | 93.42 | 43.65 | 99.2 | 90.1 |  |
| Mtrp+AH_5 | 25.55 | 170350936 | 97.59 | 92.52 | 43.57 | 99.4 | 90.7 |  |
| CTS+AH_4 | 23.95 | 159675988 | 97.48 | 92.15 | 43.62 | 99.7 | 90.5 |  |
| CTS+AH_5 | 25.22 | 168129846 | 97.66 | 92.69 | 42.98 | 99.6 | 91.5 |  |
